# The Impact on Vergence Parameters After Smartphone Gaming

**DOI:** 10.22599/bioj.335

**Published:** 2024-05-06

**Authors:** Vishal Biswas, Roshni Majumder

**Affiliations:** 1 Department of Optometry, School of Allied Health Sciences, Noida International University, India; 2 M. Optom, India

**Keywords:** Vergence, Binocular Vision, smartphone gaming

## Abstract

**Aims:**

To evaluate the impact of smartphone gaming on the vergence system of the eye.

**Settings and Design:**

A 5-month (from March 2023 to August 2023) comparative and experimental research was conducted.

**Materials and Methods:**

Eighty-two participants with a mean age of 21.98 ± 2.26 years were present in the study. Prior to assessing accommodation and vergence system characteristics, participants underwent a comprehensive eye examination. The participants were asked to play a shooting game on a smartphone for 30 minutes at a 40 cm distance. Measurements of the vergence parameters were taken before and after the activity and afterwards were compared.

**Statistical Analysis:**

Non-parametric tests were used to compare pre- and post-task measurements. The Wilcoxon signed-rank test was used to compare the variables: Negative fusional vergence (NFV), Positive fusional vergence (PFV), Near point of convergence (NPC), and Vergence Facility (VF), with the alpha error set at 5%.

**Results:**

The mean age of the participants was 21.98 ± 2.26 years. Post-task, the vergence parameters: NPC (p < 0.001), NFV for near distance (p < 0.001), PFV for near distance (p < 0.001), and VF (p < 0.001) showed significant decrease in vergence parameters.

**Conclusions:**

The study shows smartphone gaming for 30 minutes affects the vergence system, leading to binocular vision anomalies in young individuals.

## Introduction

The use of smartphones has grown in popularity globally. These devices provide many values in daily life. These include improved communication, serving as portable all-in-one devices that combine a phone, camera, GPS, and MP3 player, and additionally provide help for those with disabilities by offering verbal or motion-activated applications. (Kim et al. 2017). According to a recent report by Lee et al. (2016), 14% of middle school children had a significant risk of smartphone addiction. Similarly, a study by Choi et al. (2015) showed that 45% of the participants felt anxiety when they were asked to not to use their smartphones. Another study by Kwon et al. (2016) and Moon et al. (2014) found that excessive smartphone usage has been found to cause headache, neck ache, eye pain, blurred vision, and dry eye. Computer vision syndrome (CVS) is a term used to describe asthenopia due to prolonged use of computer screens, including those on smartphones; it manifests as symptoms of visual, ocular, and musculoskeletal pain, particularly in the neck and shoulders (Al Rashidi & Alhumaidan 2017; Blehm et al. 2005; Rosenfield 2011).

Studies by Ha et al. (2014) and Phamonvaechavan (2017) reported how accommodation and vergence system responses reduce after using a smartphone for reading activities. We carried out a comparative and an experimental study to examine how vergence parameters can change after a period of smartphone gaming. We hypothesise that an individual’s vergence parameters will reduce after the completion of the assigned task of playing games from a smartphone.

## Subjects and Methods

This comparative and experimental study was conducted within 5 months between March 2023 to August 2023. The study was approved by the Institutional Review Board and the Ethics Committee of Human Research M. Medical Centre (MMDCIRB/2023/01/002), and complies with the requirements of the Helsinki Declaration.

Participants were invited from a reputable college. Purposive sampling was utilised for sample size determination. Each participant in the research gave written informed consent before participating voluntarily.

Out of 90 participants who received an eye examination, eight were excluded for not meeting the criteria for inclusion. The comprehensive eye examination included a longer distance visual acuity test using a LogMAR (Logarithm of the Minimum Angle of Resolution) chart, with results that were later converted to the Snellen equivalent. Another eye examination for testing near vision used an English N-notation chart at 40 cm. Slit-lamp biomicroscopy was performed to assess the integrity of the ocular structures. Inclusion criteria comprised of all participants with best corrected distance visual acuity of 20/20, N6 at 40 cm using a near English reading chart, and no history of asthenopia. Those who had a squint, systemic or ocular abnormality, or a history of eye surgery were excluded. All participants gave their informed consent, and those who were excluded had their ocular conditions explained to them.

Vergence parameters were considered for analysis, including Near Point of Convergence (NPC), Negative and Positive Fusional Vergence (NFV; PFV), and Vergence Facility (VF). For the measured values acquired in this investigation, Scheiman and Wick’s (2008) standards and vergence data technique were used as a guideline (Scheiman & Wick 2008).

The subjects were seated comfortably during the activity, and a study by Padavettan et al. (2021) was used to determine the brightness level of the room. Light emitting diode (LED) lights provided the 480–500 lux brightness intensity, and window glare was removed due to the absence of windows in the room. Participants were given a Samsung A33 5G smartphone (with a 6.4-inch Super AMOLED display with 1080 × 2400-pixel display resolution and 411 ppi density) for their task. The illuminance of the display for the activity was kept constant at 420–460 cd/m^2^.

A shooting game (Shooting Archery 3.53 Version Android-based game) was chosen for this gaming activity for its intuitiveness among subjects. In this portable game, the participants had to use their bow and arrows to shoot at several moving targets, including circular targets, fruits, false targets, and many others.

The participant’s eyes were kept 40 cm away from the smartphone during the activity, and the distance was monitored every five minutes without disturbing the subject during the task using a ruler marked at the 40 cm line by the primary examiner. Within five minutes of finishing the gaming activity, vergence parameters were re-assessed.

### Tests of vergence

#### Near point of convergence (NPC)

A circular target is focused on the retina at the point nearest to the eyes, or NPC, as determined by the RAF ruler. The reliability of the findings was assessed by taking the average of three readings. The readings were noted in centimetres by Abrams (1998).

#### Negative fusional vergence (NFV) and positive fusional vergence (PFV) (distance and near)

A horizontal prism bar was used to measure the fusional vergence at both a longer distance of 6 metres and a near distance of 33 cm. A set of 20/30 letters, arranged in a column on both the distant (for assessing far fusional vergences) and near (for measuring near fusional vergences) sections of the Snellen chart, were used. Base-out (BO) and base-in (BI) prisms were used to assess the positive and negative fusional break points as well as recovery points).

#### Vergence facility (VF)

VF aims to represent the capacity of the fusional vergence system to react quickly and precisely to changing vergence demands over time. It is defined by the number of cycles per minute (cpm) that a stimulus can be fused through alternating BI and BO prisms. VF was calculated while reading N6 text at a distance of 40 cm using the prism flippers (12 Prism Dioptre (PD) BO and 3 Prism Dioptre (PD) BI).

The parameters related to the pre- and post-task were stored in separate data sheets, and pre-task values were analysed first followed by the post-tasks’ parameters to avoid experimenter bias.

### Statistical Analysis

The subject’s data was entered into a pre-designed pro forma Microsoft Excel sheet (2007). SPSS (Statistical Package for Social Sciences, Version 27.0.0 Inc., Chicago, IL, USA) was used to analyse the results. Descriptive statistics were calculated for the entire sample of participants. Non-parametric tests were used to compare data between pre- and post-task measures. The variables, NFV, PFV, NPC, and VF, were compared using the Wilcoxon Signed Rank Test, and the alpha error was set at 5%.

## Results

The mean age of the participants was 21.98 ± 2.26 years, which ranged from 18 to 30 years. Out of the participants, 30 were female (36.58%) and 52 were male (63.42%). A Titmus Fly Test showed the mean stereopsis to be 45 ± 6.13 secs/arc. Measurements ranged from 60 to 40 secs/arc. For the right eye, the mean spherical equivalent refraction was –0.71 ± 1.73 DS, and for the left, it was –0.71 ± 1.75 DS.

### Vergence parameters

#### 1. Near point of convergence (NPC)

**Figure 1:**
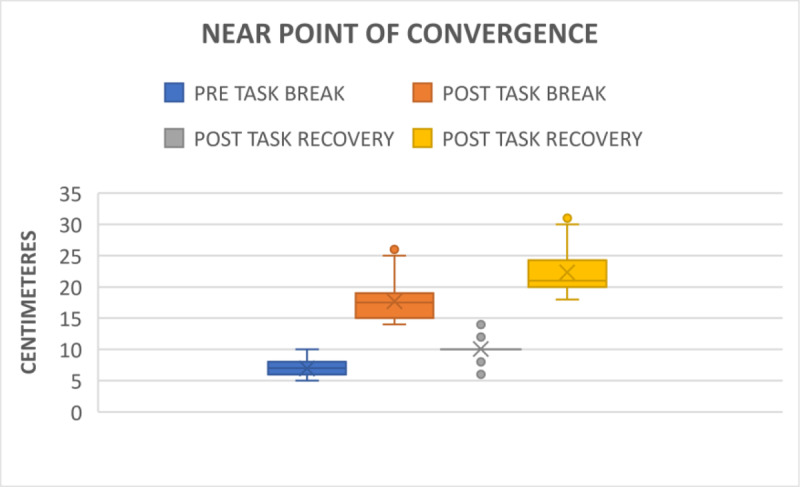
Comparison of break and recovery point of NPC before and after gaming.

The pre-task mean NPC break point and recovery point was found to be 6.97 ± 1.04 cm and 10.08 ± 1.42 cm (Figure 1) which ranged from 5–13 cm, respectively. Comparatively, after smartphone gaming, the NPC decreased to 17.70 ± 3.48 cm (p < 0.001) as the break point and 22.29 ± 3.56 cm (p < 0.001) as the recovery point (Figure 1). The post-task range of break and recovery points ranged from 14–31 cm. The result shows a negative correlation between the ability to converge after smartphone gaming. The parameters for NPC are shown in the Appendices (Table 1).

#### 2. Negative Fusional Vergence (NFV)

**Figure 2:**
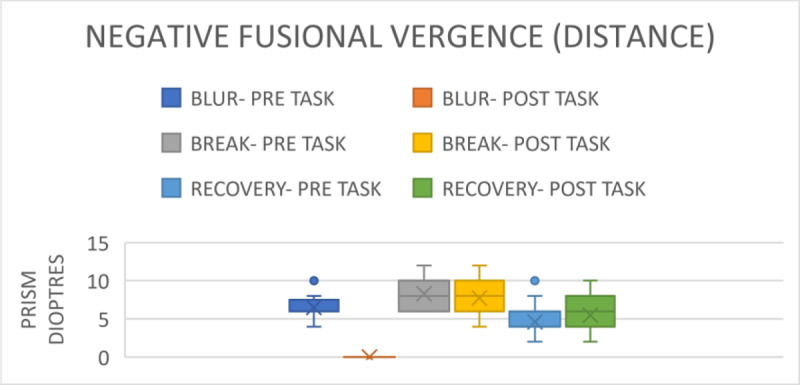
Comparison of NFV for distance before and after gaming.

For the distanced assessment, the mean pre-task blur point was 6.5 ± 1.93 prism dioptres. The break point was 8.29 ± 1.74 prism dioptres; the range for the break point was 6–12 prism dioptres, and the recovery point was 4.60 ± 1.76 prism dioptres. The range for the recovery points were between 2–10 prism dioptres, as shown in Figure 2. Post-task, the blur point was 0 prism dioptres (p = 0.02). The break point was 7.70 ± 2.20 prism dioptres (p = 0.04), with a range of 4–12 prism dioptres, while the recovery point was 5.46 ± 2.22 prism dioptres (p = 0.01), with a range of 2–10 prism dioptres, seen in Figure 2.

**Figure 3:**
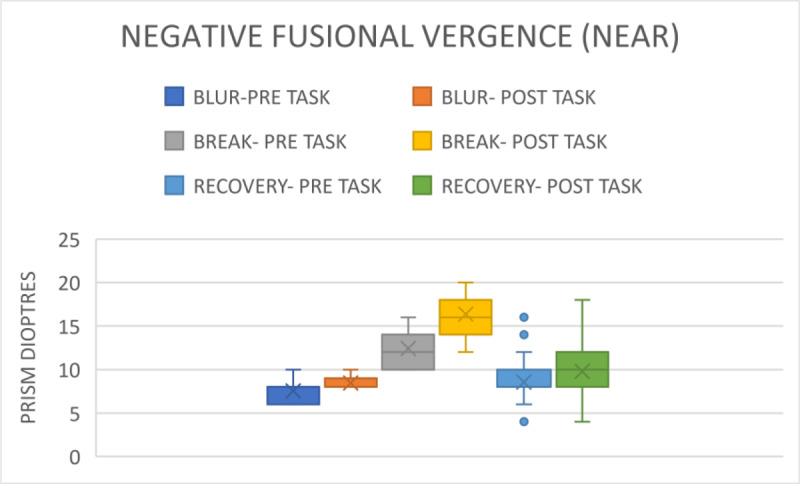
Comparison of NFV for near before and after gaming.

For the near assessment, the blur point for the pre-task was 7.53 ± 1.45 prism dioptres, with a range of 0–10 prism dioptres; the break point was 12.41 ± 2.14 prism dioptres, with a range of 10–16 prism dioptres; and the recovery point was 8.46 ± 2.13 prism dioptres, with a range of 10–16 prism dioptres (Figure 3). The post-task blur point was 8.34 ± 0.82 prism dioptres (p = 0.84). The break point was 16.36 ± 2.66 prism dioptres (p < 0.001); the recovery point was 9.80 ± 2.97 prism dioptres (p < 0.001), and the range of the recovery point was 4–18 prism dioptre visualised in Figure 2. The parameters are shown in the Appendices (Table 1).

#### 3. Positive Fusional Vergence (PFV)

**Figure 4:**
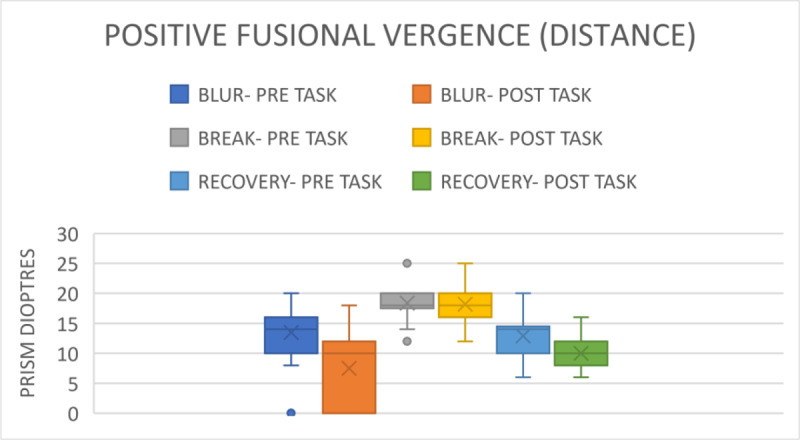
Comparison of PFV for distance before and after gaming.

Following the distanced assessment, the pre-task blur point was 13.48 ± 4.00 prism dioptres. The break point was 18.92 ± 2.05 prism dioptres, and the recovery point was 12.97 ± 2.93 prism dioptres (Figure 4). The range for all three was 6–20 prism dioptres. Post-task, the blur point was 7.39 ± 6.26 prism dioptres with a range of 2–14 prism dioptres; the break point was 18.19 ± 3.07 prism dioptres with a range of 12–25 prism dioptres (p = 0.05), and the recovery point was 10.02 ± 2.09 prism dioptres with a range of 6–16 prism dioptre (p < 0.001).

**Figure 5:**
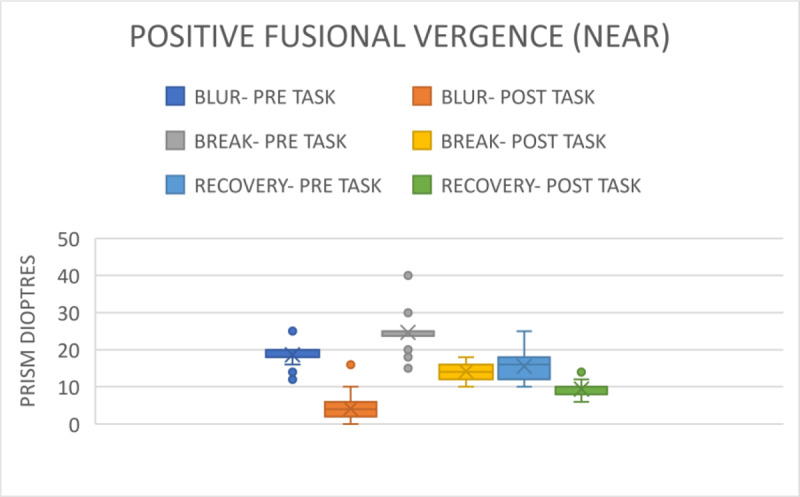
Comparison of PFV for near before and after gaming.

**Figure 6:**
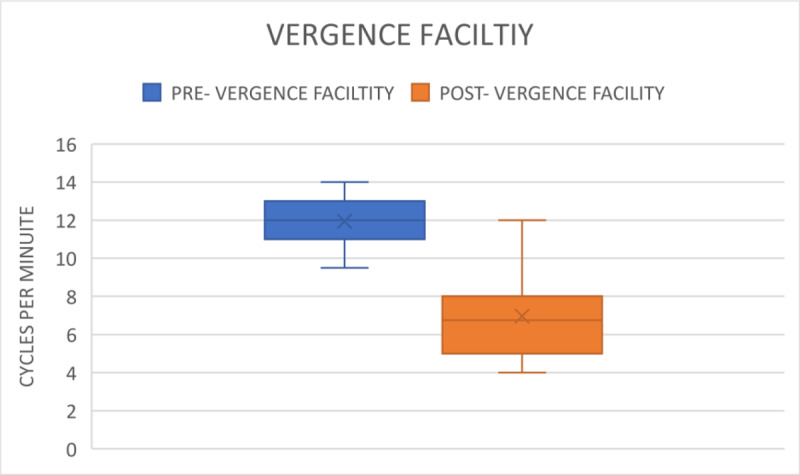
Comparison of Vergence Facility before and after gaming.

The blur point for the near distance pre-task vision assessment was 16.62 ± 6.04 prism dioptres; the break point was 25.67 ± 3.49 prism dioptres, and the recovery was 15.63 ± 3.75 prism dioptres as (Figure 5). The ranges for recovery were between 6–20 prism dioptres. Post-task, the blur point was 4.21 ± 3.04 prism dioptres with a range of 2–6 prism dioptres. The break point was 14.26 ± 2.12 prism dioptres with a range of 10–18 prism dioptres (p < 0.001); and the recovery point was 9.29 ± 1.81 prism dioptres with a range of 6–14 prism dioptres (p < 0.001) (Figure 6). All the parameters are visible in Table 1 in the Appendices.

#### 4. Vergence Facility (VF)

The mean VF before the game task was 11.93 ± 1.20 cpm. After task completion, it reduced to 6.96 ± 2.17 cpm, ranging from 4–12 cpm (p < 0.001) as visualised in Figure 6. This further provides evidence for reduced ability to converge and diverge in one minute. The VF parameters is shown in Table 1 in the Appendices.

## Discussion

Our study found a significant decrease in vergence parameters after 30 minutes of continuous smartphone gaming. Padavettan et al. (2021), Kwon et al. (2016) and Lee et al. (2016) reported that worsened NPC might lead to asthenopia while performing prolonged near visual tasks. We additionally found a significant worsening of NPC break and recovery points after the smartphone gaming task. This suggests that prolonged smartphone gaming might result in reduction in the ability to converge, which may increase the risk of ocular fatigue. The current study is in keeping the existing reports which are measured by Long et al. (2017) and Park et al. (2014). This implies that after a prolonged period of smartphone gaming,, the probability of asthenopia increases.

Furthermore, NFV and PFV showed significant decreases after gaming. The results indicate that smartphone gaming leads to a reduction in fusional and accommodating vergence. Consequently, the participants require sufficient reserve in both parameters after prolonged smartphone gaming to regain binocularity. If not, individuals risk developing the asthenopic symptoms as described in trials by Long et al. (2017) and Park et al. (2014). After the near distanced visual activity of watching a movie on a smartphone for 30 minutes, Park et al. (2014) found a substantial decrease in NFV in both groups with and without presbyopia. Similarly, Padavettan et al. (2021) reported in a study that NFV and PFV strongly worsened after 30 minutes of reading activity, which is comparable to the 30 minutes of smartphone gaming that we found in our study. According to Phamonvaechavan (2017), reading content on a 9.7-inch screen sized iPad with a 50-cm liquid crystal display for 20 minutes reduces fusional convergence in individuals under the age of 30. However, this finding was not replicated when smartphones were used by people of the same age for 30 minutes at a distance of 40 cm, according to Kwon et al. (2016). It is also important to note that our study’s post-task VF measures showed a significant worsening, from 11.93 ± 1.20 cpm to 6.96 ± 2.17 cpm.

These findings elucidate the impact on vergence parameters in binocular vision after 30 minutes of non-stop smartphone gaming. Though, it would have contributed more information on this experiment to compare the accommodative findings and stereopsis.

## Conclusion

Playing smartphone games for 30 minutes has a noticeable impact on the vergence system and its related components when doing near distance visual tasks. Long exposure to smartphones may cause binocular vision anomalies and ocular fatigue considerably early in young individuals. Therefore, taking regular breaks when reading on a smartphone is advised. However, further research is needed to comment on the impacts smartphone gaming can have on the accommodative system of the eye.

## Appendices

**Table 1:** Vergence parameters mean and standard deviation (SD) for the pre- and post-task activity (Wilcoxon Signed Rank Test).

PARAMETERS		PRE-TASK		POST-TASK		P-VALUE
	
		MEAN	SD	MEAN	SD	

NPC	Blur	6.97	1.04	17.70	3.48	<0.001

	Break	10.08	1.42	22.29	3.56	<0.001

NFV (D)	Blur	6.5	1.93	0	0	0.02

	Break	8.29	1.74	7.70	2.20	0.04

	Recovery	4.60	1.76	5.46	2.22	0.01

NFV (N)	Blur	7.53	1.45	8.34	0.82	0.84

	Break	12.41	2.14	16.36	2.66	<0.001

	Recovery	8.46	2.13	9.80	2.97	<0.001

PFV (D)	Blur	13.48	4.00	7.39	6.26	0.06

	Break	18.92	2.05	18.19	3.07	0.05

	Recovery	12.97	2.93	10.02	2.09	<0.001

PFV (N)	Blur	18.62	3.04	4.21	3.04	<0.001

	Break	25.67	3.49	14.26	2.12	<0.001

	Recovery	15.63	3.75	9.29	1.81	<0.001

VF		11.93	1.20	6.969	2.17	<0.001
